# Big advocacy, little recognition: the hidden work of Black patients in precision medicine

**DOI:** 10.1007/s12687-023-00673-9

**Published:** 2023-09-29

**Authors:** Lynette Hammond Gerido, Kenneth Resnicow, Elena M. Stoffel, Tiah Tomlin, Robert Cook-Deegan, Melissa Cline, Amy Coffin, Jill Holdren, Mary Anderlik Majumder, Zhe He

**Affiliations:** 1 Department of Bioethics, School of Medicine, Case Western Reserve University, 10900 Euclid Ave, Cleveland, OH 44106-4976 USA; 2 School of Public Health, University of Michigan, Ann Arbor, MI USA; 3 Rogel Cancer Center, University of Michigan, Ann Arbor, MI USA; 4 Patient Advocate, Atlanta, GA USA; 5 Arizona State University, Washington, DC USA; 6 University of California Santa Cruz, Santa Cruz, CA USA; 7 Virginia Commonwealth University, Richmond, VA USA; 8 Patient Advocate, Boulder, CO USA; 9 Baylor College of Medicine, Houston, TX USA; 10 Florida State University, Tallahassee, FL USA

**Keywords:** Breast cancer, Genetic testing, Advocacy, African Americans, Biomedical research, Data ethics

## Abstract

As cost-effective next-generation genome sequencing rapidly develops, calls for greater inclusion of Black people in genomic research, policy, and practice are necessary for effective translation of genomic science into precision population health and medicine. Employing a community-based participatory mixed methods research design, we developed a semi-structured survey that was disseminated to three cancer advocacy organizations. Of the 81 survey respondents 49 (60%) self-identified as Black, and 26 (32%) indicated a prior breast cancer diagnosis. Black participants’ expressed concerns about genetic testing were evenly distributed between concerns that could be addressed through genetic counseling (24%) and concerns about subsequent use of their genetic data (27%). Patient advocates contributed to contextualization of respondent concerns in terms of community experiences. Although genetic counseling services and policies governing genomic data use are not always accessible to many Black communities, advocates on our research team provided a bridge to discussion of the intersection between respondent concerns and the roles advocates play in filling gaps in access to genetic counseling and data governance. Concerns expressed by Black patients underscore a shared need among all patients for access to education, inclusion in research, and assurances regarding the use and handling of genetic data. Black cancer patients have joined in patient-led efforts to overcome systemic inequities in cancer care to improve their health outcomes through representation. Often their efforts are overshadowed by a relentless burden of continued health disparities. Future research should support their hidden work as a means to reduce barriers and improve representation in genomic databases.

## Introduction

Public health genomics prioritizes the effective and ethical translation of genomic science into population health benefits and personalized health care. Genetic testing is often the first step in the path to precision population health and medicine. However, many cancer patients face barriers to accessing genetic testing and counseling services (Khan et al. 2022), and Black cancer patients have more pronounced disparities in survival. Disparities will widen (Huey et al. 2019) and personalized medicine cannot reach its full promise if barriers prevent many from benefiting.

Genetic testing has been available for many hereditary cancers since the 1990s. For Black patients in the USA, hereditary breast, ovarian, and colorectal cancers highlight stark disparities. Breast cancer incidence rates are higher among younger (<45 years old) Black women than among white women and mortality from breast cancer is 42% higher in Black patients than in white patients. Similarly, colorectal cancer tends to present earlier in Black patients than in white patients and Lynch syndrome is the most common colorectal cancer syndrome among high-risk Black patients (Garland et al. 2021). Even so, Black hereditary cancer patients are less likely to be referred to genetic testing and counseling services (Chapman-Davis et al. 2021; Dharwadkar et al. 2022; Sheppard et al. 2014).

Many interventions have been developed to address the disparity in breast cancer mortality between Black and white patients (Copeland et al. 2018), and Black breast cancer survivor-advocates play a critical role in addressing disparities (Jackson et al. 2021; Lythcott et al. 2003). Advocacy, which includes providing different types of support, improves self-advocacy and health-protective behaviors for the networks in which Black patients are embedded (Molina et al. 2016). Therefore, the goal of this paper is to explore the concerns and experiences of Black breast cancer patients related to genetic testing for hereditary breast and ovarian cancer (HBOC) and to describe how those concerns relate to the hidden work of patient advocates.

## Method

### Study design

The study that is a basis for this paper employed a community-based participatory mixed methods research design. It was conducted in partnership with three breast cancer advocacy groups My Breast Years Ahead, a support group created by Black women to support others with breast cancer and affiliated with My Style Matters; Brave Bosom, a citizen data science organization affiliated with The Light Collective; and Facing Our Risk of Cancer Empowered (FORCE), a nonprofit organization for people with hereditary breast, ovarian, and other cancers. The first author conducted extensive pre-study fieldwork by attending meetings held by the advocacy groups and volunteering locally at events hosted by the Florida Breast Cancer Foundation (FBCF). Two other authors hold leadership roles within breast cancer advocacy groups [TT and JH] and were a critical bridge to understanding concerns of HBOC patients throughout the study. As a result of the fieldwork, we determined our research priority would be to better understand racial differences in concerns about genetic testing for HBOC. The research was conducted in Tallahassee, Florida, as part of a PhD dissertation in Information Science. 

### Sample

Adult participants, aged 18 or older, were recruited through snowball sampling. Black women with breast cancer were deliberately targeted for recruitment. An invitation to participate in the study, including an anonymous link to the Qualtrics secure online survey software, was distributed on social media outlets and online via cancer advocacy groups’ web posts or newsletters, and was posted on social media sites with messaging to encourage readers to share the invitation with others. Cancer advocacy groups organized around inherited cancer risk constituted a natural constituency. Surveys were considered complete if more than 80% of the eligible questions were answered. For their time and effort, all participants who completed the survey received a $10 Amazon gift card.

### Data collection

The data were collected using a secure online survey, which included structured items and unstructured free text questions, adapted from validated measures in the Health Information National Trends Survey (HINTS) (*Health Information National Trends Survey | HINTS*, n.d.). The National Cancer Institute’s HINTS is a nationally representative cross-sectional survey administered biennially to adults aged 18 years and older in the USA to monitor health information awareness and communication (Hesse et al. 2017). We refined the instrument with input from the advocates to include an open-ended qualitative question about genetic testing concerns. Data collection took place between March and November 2020. Both quantitative (structured) and qualitative (unstructured, free text) data were collected.

### Measures

We collected the following socio-demographic information: self-reported race, ethnicity, age, education, income, marital/parental status, insurance, and city of residence. Cities where the participants currently reside were grouped into geographic regions. Questions to capture a brief health history (self-reported history of a breast cancer diagnosis and family cancer). We collected information about the participants’ awareness of genetic tests in general and their past experience with genetic testing. In addition, participants were asked about their willingness to take a genetic test and about genetic counseling. Genetic testing concerns were captured as an unstructured field, in which participants were asked to type a response to the question “Do you have any concerns about genetic testing? Please describe.” This paper focuses on the qualitative data; quantitative data are reported in the published dissertation.

### Data analysis

Prior to analysis, all responses were deidentified and assigned a unique participant number. We conducted a complete case analysis to screen for errors, determine frequencies, and identify normality of distribution patterns in the data to assess its overall quality. We used R to conduct exploratory data analysis and preprocess the data.

Descriptive statistics were calculated for all variables of interest. Continuous measures were summarized using means, standard deviations, and ranges. Categorical measures were summarized using counts and percentages.

### Thematic analysis

We created a preliminary unstructured coding guide for unstructured, free text responses based on constructs of Dervin’s Sensemaking Theory (Dervin 1998). Sensemaking theory provides a framework for identifying situations, recognizing barriers and social contexts, describing gaps or discontinuities, and creating bridges to desired outcomes. We introduced six undergraduate research assistants (URAs) to coding unstructured data using the coding guide. During a weekly lab meeting the URAs and the first author reviewed 10 random responses to the unstructured question capturing genetic testing concerns. Line by line data coding was then conducted collaboratively to assist in clustering of responses and refining the codebook. As the URAs reviewed each response, they associated it with items in the preliminary coding guide or refined the guide with new codes as required. Codes were compared and discussed until we arrived at a consensus. The final code structure contains 8 codes, which we subsequently categorized under two overarching themes: concerns that could be addressed with genetic counseling and concerns about subsequent use of genetic information. Each code or sub-theme was described in collaboration with our partners to further contextualize our findings. Both the sub-theme descriptions and advocate contextualizations are included in the “Results” section.

## Results

### Participant sociodemographic and background characteristics

Out of 102 surveys submitted, 81 were complete. Participants ranged in age from 24 to 76 years. All resided in North America. The sample was stratified by race (Black, Asian, White, and Other). Forty-nine respondents (60%) self-identified as Black. Twenty-six (32%) indicated they had a history of a breast cancer diagnosis.

All participants identified their gender as female or gender nonconforming. The study population was educationally skewed, with higher educational achievement than the general population (18.5% with high school diplomas, some college, or an Associate’s degree; 30.9% with a Bachelor’s degree; and 48.1% with a Master’s degree or higher). The study population reported 28.4% below $60,000, 34.6% between $60,000 and $99,000, and 27.2% with $100,000 or more annual household income. Most had insurance (93.8%), were married (48.1%), and had children (76.5%). Only 8.6% indicated Latinx or Hispanic ethnicity. Black participants represented 60.5% of the study population.

### Racial differences in concerns

The frequency of the two themes (concerns that could be addressed with genetic counseling and concerns about subsequent use of genetic information) between Black participants and non-Black participants showed interesting differences (Table 1). Nearly half (49%) of the Black participants expressed no concerns with genetic testing, in comparison to fewer non-Black participants who expressed no concerns (28%). Black participants with concerns were fairly equally distributed between concerns that could be addressed with genetic counseling (24%) and concerns about subsequent use of their genetic data (27%).

**Table 1:** Comparison of themes by race

	No concerns	Concerns that could be addressed with genetic counseling	Concerns about subsequent genetic data use
Black (*n*=49)	24 (49%)	12 (24%)	13 (27%)
Non-Black (*n*=32)	9 (28%)	15 (47%)	8 (25%)
All respondents (*n*=81)	33 (41%)	27 (33%)	21 (26%)

Non-Black participants shared more concerns that could be addressed with genetic counseling (47%) than concerns related to subsequent use of their genetic test data (25%). Both Black and non-Black participants expressed similar concerns, as they are described in the following passages, which include exemplar quotations from surveys and contextual information shared by advocates. This contextual information links survey responses to lived experience, including learning from efforts to help Black women, in particular, navigate health care and other systems.

## Theme I: concerns that could be addressed with genetic counseling

There were five unique concerns expressed by participants that traditionally have been addressed by genetic counselors: validity of results, medical decisions, education, insurance considerations, and family communications.

### Validity of results

Participants reported concerns about the validity of genetic test results. For example, one participant expressed concern about:

“possibilities of inaccuracies”

(Participant #3: Black/Age 43/No - Breast Cancer Diagnosis)

#### Advocate contextualization

Along with validity, patients seek certainty from genetic test results. Yet approximately 1 in 3 Black breast cancer patients undergoing germline genetic testing with a multigene panel is found to carry one or more variants of uncertain significance (VUS); VUS results; lack both the clinical actionability of a pathogenic variant and the reassurance of a benign/negative result. Populations with African ancestry are underrepresented in genetic databases used to classify variants, which reflects the continued need for discovery to improve the clinical validity of genetic testing results.

### Medical decisions

Genetic testing results are important tools for making medical decisions. At various stages along the breast cancer care continuum, patients can feel unsure how to make decisions based upon the results of the genetic test, as expressed by this participant:

“My concern is when they find an anomaly and there isn’t any information in how to deal with it.” (Participant #50: Black/Age 44/Yes - Breast Cancer Diagnosis)

#### Advocate contextualization

Some patients may opt to be tested before breast cancer is suspected because they have a family history of HBOC or perhaps they engaged in recreational testing purely for the knowledge. Others may be recently diagnosed with cancer and need to use the information to make surgical decisions to prevent the progression of the disease. Still others may have survived cancer and have incorporated genetic testing as a means of surveillance in case of recurrence. In each of these situations, the genetic testing would address and inform (or fail to inform, as some participants feared) different types of medical decisions.

### Education

Participants expressed a need to be educated about the process and what to expect after the testing has been completed. For example:

“Honestly I probably don’t know enough about the testing to really know if I should be concerned.” (Participant #65 - Black/Age 64/Yes - Breast Cancer Diagnosis)

#### Advocate contextualization

Patients may need information in order to raise questions and self-advocate.

### Insurance

Insurance concerns were expressed by the participants. For example, they expressed worry about:

“Insurance ramifications”

(Participant #8 - Black/Age 37/No - Breast Cancer Diagnosis)

#### Advocate contextualization

These types of concerns may be extremely important for marginalized groups due to increased likelihood of encountering structural barriers to care and limited insurance benefits. Some patients may be reluctant to agree to genetic testing due to perceived changes in medical costs or reduced insurance coverage.

### Family communications

When patients receive the news that they have tested positive for a hereditary cancer syndrome, it can impact the entire family. Patients who have a pathogenic variant often carry the primary responsibility to communicate directly with relatives, which can be especially difficult for parents. For example, parents may be unsure when to discuss testing with their children, as noted by this participant:

“I do [have concerns] for my own children - not sure when is too early.”

(Participant #43 - White/Age 51/Yes - Breast Cancer Diagnosis)

#### Advocate contextualization

Standard practice is for providers to support patients with “a family letter” to share the news of their genetic risk and to encourage them to seek genetic testing and counseling services as well.

## Theme II: concerns about subsequent use of the genetic information

Concerns about how the genetic information would be stored, kept private, or destroyed after testing were described. Participants wanted to know who would have access to the information and whether it would be used to socially discriminate against them or to commit genocide.

### Agency

In addition to using genetic testing results to make medical decisions, patients would like to have agency in what happens to their genetic information. For example:

“After the testing what happens to my genetic samples? Will they be kept, disposed of, or returned to me? Am I able to make that decision?” (Participant #39 - Black/Age 26/No - Breast Cancer Diagnosis)

### Privacy

Concerns about agency are related to protecting the privacy of such sensitive information. Truly protecting privacy can be a challenge even with de-identification, especially given the ease with which data flows:

“Information security, privacy, testing or any part of the data analysis being done outside the U.S.” (Partici`pant #56 - White/Age 55/Yes - Breast Cancer Diagnosis)

#### Advocate contextualization

A complete genetic sequence is unique to the individual, and even if it has been separated from other personally identifiable information, this genetic data is forever linked to the individual. Further, people often view genetic information about themselves as private. At the same time, specific variants within an individual’s genome may be widely shared with biological relatives or even across the entire human population, and data sharing has the potential to benefit relatives and advance population health.

### Third party access

Often laws limit how genetic data may be used or by whom the data may be accessed but frequently patients are not aware of how their genetic data will be protected. Participants wanted information about third party access in advance, for example:

“I learned after the test about potential problem of obtaining life insurance because of genetic testing regardless of the results. I feel those who are tested should be made aware of that fact.” (Participant #29 - Black/Age 53/Yes - Breast Cancer Diagnosis)

### Social discrimination

Some marginalized communities have faced historic trauma related to discrimination and oppression, which bleeds into current day experiences with social discrimination. Concerns may be related to secondary use such as by law enforcement and/or for commercialization, for example:

“My concern would be if my results could be used towards other testing or sold to outside companies.” (Participant #47 - Black/Age 33/No - Breast Cancer Diagnosis)

#### Advocate contextualization

Black breast cancer patients may experience disproportionate exposure to forensic surveillance and overrepresentation in law enforcement genetic databases. Consideration of the additional dimensions of discrimination may be a priority among Black patients.

### Genocide

Participants were concerned about the deliberate use of data to control, harm, or kill groups of people, as in this participant’s expression of concern about genocide.

“Just worry future Nazi genocidal maniacs will use the information against us.” (Participant #79 - Multiracial/Age 41/No - Breast Cancer Diagnosis)

#### Advocate contextualization

The historical ties between human genome research and antiquated eugenics practices may foster additional concerns that extremist organizations will use genetic information to carry out genocide.

## Discussion

Our study demonstrates the value of partnership with underrepresented communities in public health genomics research and provides a foundation for expanding awareness of patient advocacy efforts related to HBOC. When one thinks about breast cancer advocacy, typically the efforts are seen through a lens of social support. Many advocates address barriers related to social determinants of health by providing transportation to appointments, raising funds for housing, and offering healthy meals. Yet there are additional collective advantages of having advocacy groups embedded within research teams. Advocacy extends beyond clinical research settings into underrepresented communities. From the outset of this study, partners from underrepresented communities affected by cancer and specifically those that supported Black breast cancer patients were engaged. This aided in successfully representing Black women in our study population.

One contribution of our study is adding nuance to accounts of medical mistrust by unpacking patient concerns specific to genetic testing. Schumann et al. (2021) found that medical mistrust may articulate critique of the profit-making with patient data (Schumann et al. 2021). Many commentors reference the historical traumas from the infamous Tuskegee Syphilis study to provide critical context for medical mistrust. However, these types of framings are overly simplistic if they fail to account for many modern-day information technology concerns. We uncovered two major categories of concerns expressed about genetic testing, one reflecting a need for genetic counseling prior to testing and the other reflecting issues specific to genetic data use after testing. Although Black participants in this study raised concerns about genetic testing that are similar to those raised by non-Black participants, their data use concerns highlight an important need. We must increase access to genetic counseling services in parallel with more transparent disclosures and assurances about the use and handling of genetic data. These findings should be viewed in context with existing patient-led efforts to overcome systemic inequities in cancer care. Advocacy groups have become an ongoing source of emotional support and a bridge to activism and representation (Braun 2003). Black breast cancer patients have been partnering with advocates and researchers to educate communities, develop protective health data initiatives, and to improve the representativeness of genomic data for quite some time.

### Genetic counseling and education

Our study shows that among Black participants who expressed concerns about genetic testing, nearly half of those concerns could be addressed by having access to genetic counseling services. Typically, genetic counseling helps patients make informed, autonomous choices about whether or not to undergo a genetic test, how to interpret the results, and how to weigh options related to the results. Medical decisions can be challenging, and genetic testing can increase the complexity of considerations. For example, advocates can share information about the Genetic Information Nondiscrimination Act of 2008 (GINA). GINA prevents health insurance plans from discriminating against patients with genetic susceptibility for diseases and disorders. Individuals considering genetic testing also need to know that GINA does not prevent discrimination in life, disability, and long-term care insurance, although some state laws may help to fill these gaps. In addition, advocates have developed extensive educational materials to describe the differences between direct-to-consumer (DTC) genetic testing not regulated by Clinical Laboratory Improvement Amendments (CLIA) (i.e., 23andme) and genetic testing provided by laboratories regulated by CLIA (i.e., Color Health). These educational materials are designed to help patients understand the differences in testing technologies, the limitations of the results, and the potential data risks related to the subsequent use of their genetic data. This work is significant for Black breast cancer patients, who may require CLIA-certified genetic testing because it employs comprehensive sequencing to identify more than the three Ashkenazi Jewish founder variants in BRCA1 and BRCA2 assessed by some DTC genetic tests. Also, relevant to patient concerns about data use, CLIA-certified laboratories are bound by patient privacy regulations and are not able to resell genetic testing data. Educational materials about these genetic testing options are accessible to supplement information from health care providers. This is an example of information that would be discussed as a part of genetic counseling and shared through advocacy networks.

Unfortunately, there is a clear shortage of health professionals specifically trained to provide genetic counseling (Hoskovec et al. 2018). Cragun and colleagues observed that only half of young Black women with breast cancer who were eligible were referred to accessed genetic services despite national practice guidelines (Cragun et al. 2015). Chapman-Davis and colleagues reported significant differences in referral patters for genetic counseling (Chapman-Davis et al. 2021) and Sheppard and colleagues reported increased patient satisfaction among Black women who received in-person support to discuss treatment options for breast cancer (Sheppard et al. 2010).These examples expose voids and unmet needs that advocacy groups fill for patients lacking access to genetic counseling. Without access to certified genetic counselors, many patients facing a hereditary cancer diagnosis seek information and decision support from trusted advocacy groups (Davis and Baca 2015). Many advocacy groups like My Breast Years Ahead disseminate educational materials on behalf of healthcare providers and direct vulnerable patients to additional resources. Advocacy groups also assist patients in improving their communicative competence with providers and their family members.

### Data use and governance

Our findings indicate that Black participants are interested in genetic testing, but they have reservations about the misuse and abuse of genetic data. The technologies and data structures, which support clinical genetic services must be evaluated and updated often. Recent news headlines describing the racial inequities built into clinical algorithms used for monitoring and surveillance can erode trust in clinical technologies. Scholars have noted, “…we are in need of new analytic tools, forged by critical scholars of science and technology, that draw into view the mutual constitution of social and scientific practice”(Reardon 2008). We must reimagine equity and justice in healthcare and recognize that systems architectures can engineer inequality (Benjamin 2016). We can reconnect with the efforts of geneticists, like Mary-Clare King, whose activism and social justice agenda bridged scientific and sociopolitical spheres (Nelson 2016). Such a re-imagining should take seriously concerns about the use of data to control or harm marginalized social groups. As noted above, one participant expressed concern about genocide, the deliberate killing of people related by geographic region, ethnicity, religion, national origin, or other physical or genetic characteristic. This may sound farfetched, but there were a number of genocidal acts in the twentieth century, including the Holocaust. The kinds of surveillance enabled by modern information technology increase the potential for misuse.

Communities, patients, and advocates need to be embedded within interdisciplinary teams that identify variants and build systems that use their data. Co-creation of knowledge and systems is required to build and sustain trust with diverse communities. Data trusts may facilitate the use of data across organizational boundaries and protect the interests of multiple stakeholders (Gomer and Simperl 2020) by empowering patients with control over their personal data (Raab 2021). The Light Collective is an advocacy group that works with breast cancer and other online patient peer support communities to develop models of collective data governance where patients control how their data are used, by whom, and for what purpose. Future research should offer opportunities for advocates to engage in research intended to govern the subsequent use of genetic information as well as offer adequate financial support and resources for existing educational outreach activities. We must recognize the invisible labor of breast cancer advocacy groups that currently serve the underserved and underrepresented communities.

### Strengths and limitations

Initiatives to engage underrepresented populations in cancer clinical trials and genomic research are growing (Ramirez and Thompson 2017; Saulsberry and Olopade 2021). Our study design emphasized the value of patient partnership throughout the study. The results add to the literature that intersects precision medicine and sociotechnical systems to advance medical care and reduce health disparities. Although physician referrals, patient education, and genetic counseling are all important, sociotechnical experiences should be considered as well. Therefore, a strength of this study is presenting a sociotechnical lens of genetic testing concerns through patient narratives. Patient narratives allow participants to share their lived experiences and personal perspectives to enrich our understanding of barriers to genetic testing. At the same time, our study was limited in scope and used a small purposive sample. Future research should be conducted with a larger, less homogenous sample to verify the types of concerns, document the frequency at which they are present and generalizable in the population, and identify discordance within and between groups.

## Conclusion

Community partnered approaches to addressing racial health disparities in precision population health and medicine are required to improve access to genetic testing and hereditary cancer outcomes. Data security and privacy protections are a priority. Both traditional genetic counseling and protective data initiatives are required to educate and build trust with underrepresented communities. Fostering meaningful partnerships with breast cancer advocacy groups—and recognizing the value of their work—may be the key to achieving representative genomic datasets and more equitable access to precision medicine.

## Data Availability

The de-identified and redacted data are available from the corresponding author upon reasonable request.

## Declarations

This study was reviewed and determined by Florida State University to be exempt due to less than minimal risks of participation. All procedures performed with human subjects were in accordance with the principles of the 1964 Declaration of Helsinki and its later amendments or comparable ethical standards. Informed consent was obtained from all participants for being included in the study.
